# The Application of the Vesikari and Modified Vesikari Severity Scores in Complicated Pediatric Gastroenteritis of Viral Origin: An Observational Study

**DOI:** 10.3390/jcm14030943

**Published:** 2025-02-01

**Authors:** Maria Oana Săsăran, Cristina Oana Mărginean, Carmen Viorica Muntean, Ana Maria Pitea, Lidia Man, Alina Grama, Ana Maria Koller

**Affiliations:** 1 Department of Pediatrics 3, “George Emil Palade” University of Medicine, Pharmacy, Sciences and Technology of Târgu Mureș, Gheorghe Marinescu Street No 38, 540136 Târgu Mureș, Romania; oanam93@yahoo.com; 2 Department of Pediatrics 1, “George Emil Palade” University of Medicine, Pharmacy, Sciences and Technology of Târgu Mureș, Gheorghe Marinescu Street No 38, 540136 Târgu Mureș, Romania; duicucarmen@yahoo.com (C.V.M.); miriamarilia@yahoo.co.uk (A.M.P.); lidia.man@gmail.com (L.M.); alinagrama24@yahoo.com (A.G.); 3 Doctoral School, “George Emil Palade” University of Medicine, Pharmacy, Sciences and Technology of Târgu Mureș, Gheorghe Marinescu Street No 38, 540136 Târgu Mureș, Romania; kolleranamaria@gmail.com

**Keywords:** gastroenteritis, child, rotavirus, adenovirus, Vesikari score, modified Vesikari score

## Abstract

**Background/Objectives:** Viral gastroenteritis can have a potentially fatal outcome at young ages and the recognition of severe cases could be aided by clinically derived severity scores. **Methods:** This observational study intended to conduct a comparative assessment of the utility of the Vesikari and modified Vesikari score in the evaluation of viral gastroenteritis severity and for the possible prediction of the dehydration degree. A total number of 113 children diagnosed with gastroenteritis were retrospectively enrolled and divided based on viral etiology into group 1 (34 children with unknown viral etiology), group 2 (60 children with rotavirus) and group 3 (19 children with adenovirus). **Results:** The highest mean Vesikari and modified Vesikari scores were found in group 2 (*p* < 0.01; *p* = 0.01). A significant increase in liver enzymes was also identified in patients infected with rotavirus. The highest mean diarrhea, vomiting duration and body temperature were found in group 3 (*p* < 0.01; *p* < 0.01; *p* = 0.02), as well as the highest mean inflammatory markers, such as C-reactive protein (CRP; *p* = 0.01) and the erythrocyte sedimentation rate (*p* < 0.01). Significant linear associations were found between pH, bicarbonate level, base excess and the Vesikari scores, whereas urea, CRP and aspartate aminotransferase levels were associated with both severity scores. ROC curve analysis revealed a significant correlation between the Vesikari scores and dehydration degree (*p* < 0.01), with numeric cut-off values of 11.5 being proposed for the differentiation between mild and moderate gastroenteritis and 13.5 for the distinction between moderate and severe gastroenteritis. **Conclusions:** Both severity scores are useful in clinical settings, but more studies enrolling populations with various enteral infections could provide more insight into their etiology-based performance and reflection of paraclinical changes.

## 1. Introduction

Gastroenteritis represents a major health burden worldwide, especially in children under the age of 5 years [1]. This fragile age is particularly predisposed towards developing more severe diarrheic episodes, which can require hospitalization, especially in the context of concurring medical conditions [2]. Enteric viruses remain the most frequent etiological agents of acute diarrheic disease, with rotavirus remaining the leading cause worldwide of severe pediatric acute gastroenteritis, especially in children younger than 2 years [3,4]. The rotavirus vaccine can effectively protect against severe forms of illness and has been implemented in the national vaccination scheme by several European Union countries upon the advice of the World Health Organization (WHO) [5]. Still, rotavirus vaccination remains optional among multiple European countries, including Romania, with its costs covered by the legal guardians of pediatric populations.

In children vaccinated against rotavirus, host factors such as immune status, age, chronic underlying disorders and viral/bacterial co-infections seem to play an important role in the severity of gastroenteritis. Still, independently of cofounders, the accurate assessment of gastroenteritis severity could provide a clearer image of gastroenteritis outcomes. In the 1980s, the question of the development of severity scores for the classification of gastroenteritis arose, together with the development of the rotavirus vaccine, which turned out to ensure protection against more severe disease. Thus, the Vesikari score resulted, which scores, for each of the following parameters, one to three points: the maximum number of stools/day; diarrhea duration (days); the maximum number of vomiting episodes/day; vomiting duration (days); and dehydration, defined as the pre-morbid weight loss percentage and treatment (rehydration/hospitalization) [6]. The Clark score was later developed, for the same purpose, which partially uses the criteria of the Vesikari scale, such as the maximal stool number/day, the maximal vomiting episode occurrences/day, diarrhea and vomiting duration, the maximal body temperature measured and fever duration. The score employs, in contrast to the Vesikari score, the evaluation of behavioral symptoms like irritability, lethargy and seizures and their duration [7].

The Vesikari scale has since been largely used and applied in clinical settings, and a manual was published which advises its wide use and aids in the rapid calculation of the score in 2011 [8]. Since then, a modified version of the initial score has emerged, which replaces the percentage dehydration criterion with the need for future healthcare follow-ups. The conceptual idea behind the modification of the classical score consists of a more reliable use of the newly developed score in outpatient settings, which does not provide chances for the repeated weighing of patients [9]. The score has since been validated in multiple hospitals and emergency departments in Canada, the United States of America and China [9,10,11,12,13].

The main objective of this study was to assess the utility of the Vesikari and modified Vesikari score in the comparative assessment of viral gastroenteritis severity and to investigate how the two scores mirror the dehydration degree. Furthermore, the study aimed to compare the differences in paraclinical dehydration severity and the parameters of the two scoring systems, as well as of the overall score values. Secondarily, correlations between the dehydration degree, paraclinical data and each individual score value were investigated.

## 2. Materials and Methods

### 2.1. Study Population

**Figure 1:**
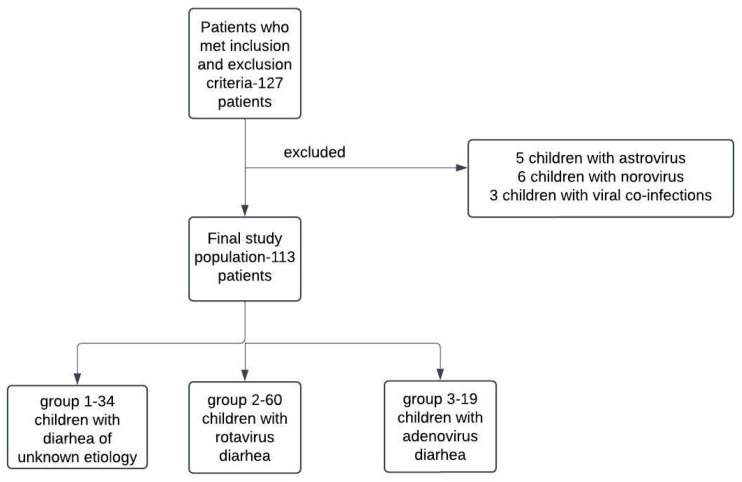
Study population enrollment and its subsequent division into study groups.

In this observational study, 113 children with viral gastroenteritis were retrospectively enrolled after applying inclusion and exclusion criteria, who were later divided into three groups. The population study consisted of children aged between 2 weeks and 12 years, who were hospitalized in a pediatric, tertiary referral center between January 2023 and September 2024. The study did not include pediatric patients with known chronic diseases which could have negatively impacted the evolution and outcome of viral gastroenteritis, such as hematologic malignancies and/or other types of malignant tumors, congenital cardiac disease, neurological disorders, chronic kidney disease, immune deficiencies and genetic syndromes. Only patients with positive viral antigen testing for rotavirus and adenovirus were included in the study, as well as those for whom the laboratory data pointed towards viral gastroenteritis, even if the rapid viral testing was negative. Patients with positive stool bacterial cultures and viral co-infections detected with rapid antigen testing, as well as those who did not require hospitalizations, were left out of the study. A more detailed selection process of the target population and its subsequent division into three study groups is detailed in the flowchart represented in Figure 1.

### 2.2. Clinical and Paraclinical Data

Clinical data were collected from the charts of the patients enrolled, focusing on the parameters of the Vesikari and modified Vesikari scoring systems as follows [6,8,10]: diarrhea duration, the maximum diarrheal stools/24 h, vomiting duration, the maximum vomiting episodes/24 h, the maximum recorded fever, the dehydration degree (assessed through the percentage of weight loss/clinical symptoms), healthcare provider visits and treatment (rehydration/hospitalization). The Vesikari score classified the illness as mild for scores lower than 7, moderate for scores ranging between 7 and 10 and severe for scores higher than 10 [6]. The modified Vesikari scale divided gastroenteritis into mild forms for scores ranging between 0 and 8, moderate for those from 9 to 10 and severe for scores of at least 11 [10]. Paraclinical data collected at the moment of admission consisted of the parameters of venous blood gas analysis (pH, Na, K, HCO_3_, base excess, lactate, glucose), the complete blood count, liver enzymes (aspartate aminotransferase—AST; alanine aminotransferase—ALT; gamma glutamyl transferase—GGT), renal function parameters and inflammatory markers (C-reactive protein—CRP, erythrocyte sedimentation rate—ESR). Stool rapid viral antigen testing involved an immunochromatography assay and tested for four frequently encountered viral etiologies: rotavirus, norovirus, adenovirus and astrovirus. Our study groups were therefore divided based on the identification of one of the four viral etiologies tested for or the absence of a known viral etiology, in cases with normal/mildly elevated inflammatory markers and negative stool cultures. Given the paucity of norovirus and astrovirus among the identified viral agents during the timeline of retrospective data collection, we excluded patients infected with these viruses in order to avoid statistical-related biases produced by small study groups. Therefore, our study population was split into a study group with viral gastroenteritis of unknown etiology (group 1), a study group with a diagnosis of rotavirus gastroenteritis (group 2) and a group with adenovirus gastroenteritis (group 3).

### 2.3. Statistical Analysis

Statistical analysis was performed with the help of GraphPad software, version 10.3.1. The significance threshold of the *p* value was 0.05, corresponding to a confidence interval (CI) of 95%. Descriptive statistics involved the delivery of parameters such as the mean ± standard deviation, median and percentage. For the assessment of data compliance to a Gaussian/non-Gaussian distribution, the Kolmogorov–Smirnov test was applied. Multiple mean comparison between numeric data were conducted with the help of the Brown–Forsythe ANOVA test for variables with a Gaussian distribution or the Kruskal–Wallis test for variables with a non-Gaussian distribution. Moreover, a separate analysis of two groups was conducted, applying the non-parameter Mann–Whitney test or the unpaired *t*-test with Welch’s correction, depending on the parameter compliance to a Gaussian distribution. A Chi-square test was applied for comparisons between categorical variables. For the investigation of the association between paraclinical parameters and the Vesikari/modified Vesikari scores, linear regression was applied. Receiver operator characteristic (ROC) curve analysis was performed with the help of the Wilson–Brown method to evaluate the performance of paraclinical parameters in relation to the two scores’ assessment of gastroenteritis severity.

## 3. Results

Our study included a population divided into three study groups, divided based on gastroenteritis etiology. The following study groups resulted: group 1—34 children with diarrhea of unknown viral etiology; group 2—60 children with rotavirus diarrhea; and group 3—19 children with adenovirus diarrhea. The mean age of the study population was 2.42 ± 2.50 years of age, while the male–female ratio was 1.89:1.

Table 1 encompasses a comparison of the baseline characteristics of the three study groups as well as of the severity of the gastroenteritis, as described through the dehydration degree, and clinical criteria which comprise the Vesikari/modified Vesikari score. No significant differences were found between the three groups in terms of age, gender or background. The prevalence of mild/moderate/severe dehydration was similar among the three groups (*p* = 0.05). However, symptoms’ duration, their maximum frequency and the value of the severity scores differed significantly between the three groups. When comparing the clinical parameters of severity between the three groups, the longest diarrhea duration could be seen in patients belonging to group 3 (2.94 ± 1.86 days vs. 0.95 ± 0.97 days and 1.45 ± 1.11 days; *p* < 0.01). In a similar fashion, the longest mean vomiting duration was found in patients infected with adenovirus (2.84 ± 1.86 days vs. 1.25 ± 0.87 days and 0.83 ± 0.56 days; *p* < 0.01). However, the mean maximum number of stools/day and mean maximum number of vomiting episodes/day were the highest among patients belonging to group 2 (*p* < 0.01). For each of these three parameters, a significant discrepancy in the mean value was seen between groups 1 and 2 and groups 1 and 3, as well as between groups 2 and 3. When analyzing body temperature, the highest mean maximum temperature was found in patients with adenovirus gastroenteritis (38.11 ± 1.17 vs. 37.42 ± 1.11 and 38.05 ± 0.99; *p* = 0.02). However, the separate analysis of two groups revealed a significant difference only between groups 2 and 1. The mean severity scores (Vesikari score/modified Vesikari score) were highest among children from group 2, reflecting a more severe clinical course of rotavirus gastroenteritis (Table 1; *p* < 0.01 and *p* = 0.01, respectively). These differences were more important when comparing adenovirus or rotavirus infections with viral gastroenteritis of unknown etiology.

Paraclinical data were also compared between the three study groups, including blood gas analysis parameters, liver enzymes, hematologic parameters, inflammatory markers and the parameters of renal function. Furthermore, each set of two groups was compared to assess mean value differences, applying the non-parametric Mann–Whitney test or the unpaired *t*-test with Welch’s correction, and the significant results (*p* < 0.05) are highlighted among the table columns as illustrated in Table 2. In terms of blood gas analysis, significant discrepancies were found in lactate levels, with the highest values among children with gastritis of undetermined viral etiology (2.42 ± 1.49 vs. 2.34 ± 2.59 and 1.37 ± 0.85; *p* = 0.01). Other parameters in the blood gas analysis did not differ significantly. The leukocyte numbers were significantly higher in group 3 (14,433 ± 4060 vs. 11,615 ± 5734 and 11,211 ± 5911; *p* = 0.03), together with the lymphocyte counts (5396 ± 2430 vs. 4019 ± 2655 and 3587 ± 3011; *p* = 0.01). Similarly, monocytes presented the highest mean values in group 3 (1406 ± 669 vs. 968.3 ± 908.7 and 1317 ± 873.5; *p* < 0.01), as was the case with eosinophils and basophils (*p* = 0.03 and *p* < 0.01, respectively). However, neutrophils presented similar values between the three groups. The reactive platelet increase was more marked in patients infected with adenovirus (488,053 ± 197,861 vs. 356,242 ± 175,028 and 420,627 ± 172,353, respectively; *p* = 0.01). The comparison of mean platelet volume yielded a significant difference, with the lowest levels in group 3 (9.18 ± 1.53 vs. 9.78 ± 1.07 and 9.31 ± 1.01; *p* = 0.02). The erythrocyte parameters were similar between the three study groups. Moreover, the highest inflammatory marker values (CRP and ESR) were found in the same adenovirus-infected group (*p* = 0.01 and *p* < 0.01). Rotavirus infection was distinguished through a higher increase in AST and ALT levels (*p* = 0.01 and *p* < 0.01). No significant differences were found between GGT, urea and creatinine levels.

**Figure 2:**
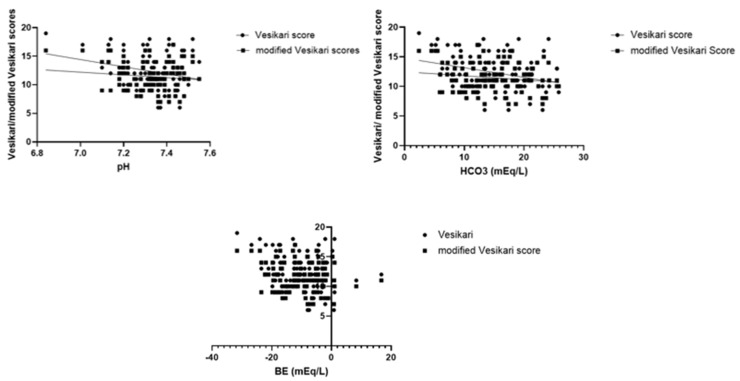
Linear regression analysis for the association between venous blood pH, HCO_3_, BE and the Vesikari/modified Vesikari scores. Legend: BE—base excess.

**Figure 3:**
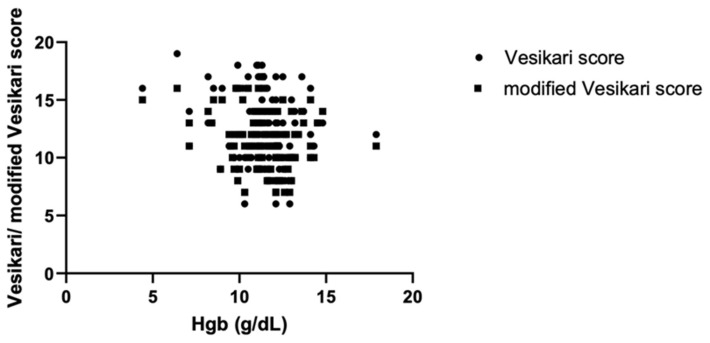
Linear regression analysis for the association between Hgb and the Vesikari/modified Vesikari scores. Legend: Hgb—hemoglobin.

Regardless of viral gastroenteritis etiology, we investigated associations between dehydration severity, paraclinical parameters and both the classical Vesikari and the modified Vesikari score (Table 3). Age did not correlate with either severity score. Dehydration severity, divided based on clinical symptoms/weight loss, correlated with both the Vesikari score (*p* < 0.01) and modified Vesikari score (*p* < 0.01), suggesting that these scores could accurately assess the gravity of dehydration. When investigating the same correlations for the parameters of the blood gas analysis, the significance threshold was reached only for the Vesikari score in the cases of the following parameters: pH (*p* < 0.01), HCO_3_ (*p* < 0.01) and base excess (*p* < 0.01). Figure 2 shows a linear decline in the Vesikari score with increasing pH and HCO_3_ and higher gastroenteritis severity scores in patients with a higher base excess (more negative values). Other blood gas analysis parameters, namely lactate, blood glucose, Na and K, did not correlate with either of the two severity scores. Multiple hematological parameters, such as leukocytes, neutrophils, lymphocytes, monocytes or platelets, were unpredictive of gastroenteritis severity, as represented through the Vesikari scores. Hemoglobin levels did, however, present a descending trend with an increase in the Vesikari (*p* = 0.02) and modified Vesikari score (*p* = 0.01). This pattern is represented in Figure 3.

**Figure 4:**
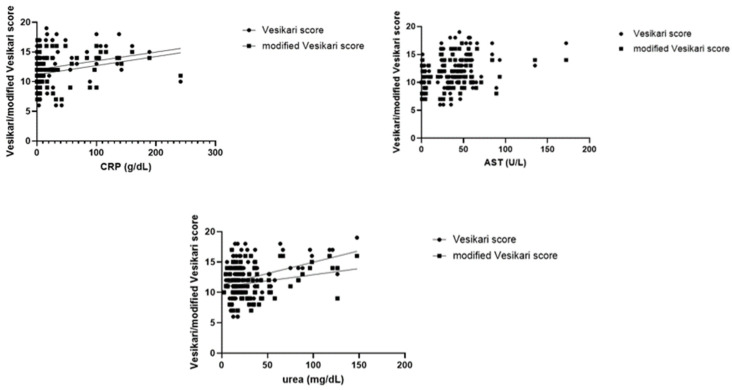
Linear regression analysis for the association between CRP, AST, urea and the Vesikari/modified Vesikari scores. Legend: AST—aspartate aminotransferase; CRP—C-reactive protein.

Increased C-reactive protein, serum AST and urea were correlated with both severity scores (*p* < 0.01 and *p* = 0.01). On the other hand, increased reactive ALT was only associated with the modified Vesikari score (*p* = 0.02). These data are represented in Figure 4. Interestingly, GGT and creatinine did not associate with either severity score.

ROC curve analysis was initially conducted to assess the ability of both severity scores to distinguish between mild, moderate and severe dehydration (as defined through the weight loss percentage and clinical symptoms), with their correspoding cut-off values. The data are provided in Table 4 and Table 5.

On one hand, the Vesikari score was able to differentiate mild forms from moderate forms of dehydration, as well as moderate forms from severe forms of dehydration, at cut-off levels of 11.5 and 13.5, respectively, but did not have high sensitivity and specificity percentages (67.35 % and 61.22% and 73.33% and 63.27%, respectively). On the other hand, the modified Vesikari score was not able to differentiate the degree of dehydration severity, as suggested by the insignificant *p* values (Table 5).

## 4. Discussion

Since the initial development of the Vesikari score for the evaluation of the rotavirus vaccine, severity scores have been used in research studies to assess gastroenteritis severity. The division of gastroenteritis forms into mild, moderate and severe forms is useful for the prediction of patients’ daycare/kindergarten/school absenteeism and the number of missed workdays for their parents [11]. The modified Vesikari score has been used as a reference for the classification of gastroenteritis in randomized, clinical, controlled trials and observational studies for the evaluation of probiotic and zinc supplementation efficacy, as well as of the importance of oral ondansetron administration at home, for reductions in gastroenteritis severity and the promotion of faster recovery from the disease [14,15,16]. Furthermore, the same score has been used as a possible predictor of clinical endpoints such as death [17].

In our study, we obtained the highest mean Vesikari and modified Vesikari scores among children infected with rotavirus, who also presented the lowest mean age (1.83 ± 1.59 years). It is well known that rotavirus infection is usually detected in children under the age of 2 years, who are hospitalized for moderate and severe forms of gastroenteritis, as determined by the Vesikari clinical severity score. Other studies have also reported that the most severe forms of gastroenteritis occur in children infected with rotavirus. Thus, a Global Enteric Multicenter Study (GEMS) reported that rotavirus infections were responsible for the highest modified Vesikari scores when compared to other viral enteric infections [18]. These results were similar to those obtained by a study from Qatar, which showed how the severity of rotavirus infection prevailed over other enteric viruses such as norovirus, adenovirus, astrovirus and sapovirus and viral co-infections [19]. Contradictorily, one study, conducted in India, which enrolled a large study population, found no significant discrepancies in the Vesikari score values between subjects with rotavirus-positive stool specimens and those without evidence of rotavirus infection. However, the study offered no information regarding the possible etiological agents in the non-rotavirus-infected study group [20].

An important influential factor of the results of the current study could be represented by a complete lack of anti-rotavirus vaccination coverage in the entire study population included. In Romania, rotavirus vaccination is not listed as part of the mandatory vaccination scheme, with its costs being optionally covered by legal guardians. Hence, there is no general recommendation for vaccination coverage against rotavirus in Romania, which unfortunately leads to severe diarrhea episodes in unvaccinated subjects, requiring hospitalizations and thus financially burdening the healthcare system [21]. The efficacy of the rotavirus vaccine in preventing hospitalizations is undebatable. One study conducted in Japan which compared a group of unvaccinated children to one of vaccinated patients, both infected with rotavirus, showed that none of the subjects of the latter group required hospitalizations and fewer required parenteral rehydration, with the same group also exhibiting fewer severe Vesikari scores [22]. However, the rotavirus vaccine might only produce a significant decrease in modified Vesikari scales in patients older than 5 years, through a significant reduction in diarrhea duration, the maximum number of vomiting episodes/day and the maximum body temperature, as suggested by a small-scale study [23].

In areas with adequate rotavirus vaccination coverage, norovirus is responsible for the most frequent possibly severe diarrhea events, with the specific mucosal immunoglobulin A antibody response not influencing the enteric infection-related symptoms [24,25]. Interestingly, it has been hypothesized that the rotavirus vaccine could reduce the severity of gastroenteritis, independently of etiology [26]. At present, in children with proper vaccination coverage, norovirus seems to trigger more severe forms of gastroenteritis. A study conducted on a Korean pediatric population vaccinated against rotavirus reported that norovirus infection, age under 2 years and possibly viral coinfection may negatively impact the evolution of acute infectious diarrhea [4]. Travel-acquired norovirus, caused by specific viral genotypes, also triggers more severe gastroenteritis episodes, with higher scores in the modified Vesikari score, than non-norovirus traveler diarrhea [27]. Unfortunately, in the current study, a separate study group with norovirus infections was not included due to the very limited number of cases, which would have been responsible for unreliable results when performing comparisons.

Within the present study, dehydration severity correlated with both severity scores when linear regression was applied. For the Vesikari score, after performing ROC curve analysis, possible cut-off levels were obtained, which could be applied for the differentiation between mild and moderate gastroenteritis, as well as between moderate and severe gastroenteritis, with satisfactory sensitivity and specificity. These results were in line with prior research showing that higher Vesikari scores can successfully identify dehydration with a high area under the ROC curve [28]. However, no significant results were obtained for the ROC curve analysis in relation to the modified Vesikari score. However, previous correlations between the dehydration degree and gastroenteritis severity, as defined by the modified Vesikari score, have been reported in other studies [10].

The current study only included gastroenteritis of viral origin, which represents a limitation. The Vesikari score seems to present higher values in infectious diarrheic episodes of bacterial origin than in similar viral infections. Hence, Shim et al. concluded that this score could be used for distinguishing the type of etiologic agent in children with acute infectious diarrhea [29]. Moreover, in order to avoid population-based biases and due to the limited number of these situations, we decided to exclude viral co-infections from our study. Still, one example of an observational study conducted in Israel reported a greater disease severity score for rotavirus and adenovirus co-infections as opposed to adenovirus alone. Similarly, the Vesikari score values were significantly higher in children infected with both adenovirus and astrovirus as opposed to individual astrovirus infections and norovirus and adenovirus co-infections, and rotavirus and astrovirus co-infections had a more adverse outcome than each individual infection [30]. These results suggest that viral co-infections are expected to require longer hospitalizations, due to a prolonged duration of symptoms and a harsher clinical picture.

Our study’s main objective was to conduct a comparison of the Vesikari/modified Vesikari clinical severity scores and their components between three study groups, diagnosed with rotavirus gastroenteritis, adenovirus gastroenteritis and viral gastroenteritis of unknown etiologies, respectively. Thus, this study allowed a comparison between two frequent etiologies of gastroenteritis in children and other viral enteric infections, while also highlighting some particularities of the adenovirus-infected group, such as the highest mean diarrhea, vomiting duration and the maximum body temperature. Adenovirus infection also produced the highest mean inflammatory marker modifications, which is in line with previous reports suggesting that this infection is responsible for the production of a systemic inflammatory response, similar to the one produced by bacterial infection, which can misguide clinicians toward the unnecessary prescription of antibiotic therapy [31]. In addition, this study aimed to investigate how the classical and modified Vesikari scores were able to reflect the dehydration degree, with significant correlations obtained between the Vesikari scores and cut-off levels provided for the distinction of the dehydration degree, after the application of ROC curve analysis. Still, the statistical analysis extended beyond a comparison of the severity scores and actually distinguished, through the integrative assessment of correlations, between paraclinical parameters of dehydration, such as those included in the venous blood analysis, markers of renal and hepatic function and the two severity scores. These bring unicity to the current study in comparison to previous studies. The results obtained also show potential discrepancies between the two severity scores. For example, pH, bicarbonates and base excess were linearly associated with higher Vesikari scores but not with the modified Vesikari score. The same parameters of venous blood gas analysis were also considerably altered with the progression of the dehydration degree in the study of Hoxha et al. [32]. Urea, which commonly increases in children with dehydration [33], and CRP correlated with both severity scores. A liver enzyme increase can mirror severe dehydration in gastroenteritis and is particularly associated with rotavirus infection, which seems to particularly infect the liver as well [34,35]. In accordance with previous studies, our results showed significantly higher AST and ALT levels in the rotavirus-infected group, and a significant association between AST and the two severity scores was obtained, as well as between ALT and the Vesikari score.

The main limitations of the current study are the number of subjects included and the constrictive enrollment of hospitalized patients. As only children who were admitted in the hospital were included, in each of the subjects enrolled, larger Vesikari and modified Vesikari scores were expected, due to the hospitalization criterion per se and the need for parenteral rehydration, besides the potentially more intense clinical picture. However, due to the retrospective character of this study and the lack of consistent follow-ups for the mild/moderate forms of gastroenteritis, which were only treated in the ambulatory and could have presented again in the emergency departments of other local hospitals, calculating the severity scores for the treated child outpatients would not have been possible. Hence, it was our decision to only include hospitalized patients.

## 5. Conclusions

The Vesikari and modified Vesikari scores represent reliable indicators of viral gastroenteritis severity in small children, which might hint toward probable etiology, according to our study. Rotavirus infection could be distinguished through the highest mean severity scores, while adenovirus was responsible for the most prolonged diarrhea and vomiting duration and the highest body temperature increase. Adenovirus triggered a higher increase in inflammatory markers than rotavirus and other enteric infections, whereas rotavirus infection led directly and indirectly to liver damage, reflected by higher liver enzymes in this group than in the other study groups. Independent of etiology, the cut-off levels of the Vesikari score could be successfully used for distinguishing dehydration levels. Future studies conducted on larger populations are warranted to provide more information regarding the performance of the severity scores for the assessment of clinical endpoints and their association with paraclinical parameters.

## Figures and Tables

**Table 1:** Comparison of patients’ clinical characteristics of diarrhea severity, based on gastroenteritis etiology.

Parameter		Group 1 (*n* = 34); *n*/Mean ± SD	Group 2 (*n* = 60); *n*/Mean ± SD	Group 3 (*n* = 19); *n*/Mean ± SD	*p* Value *
Age (years)		3.00 ± 3.01	1.83 ± 1.59	2.04 ± 1.28	0.08
Background	Urban	22	30	9	0.39
	Rural	12	27	10	
Gender	Male	20	39	13	0.61
	Female	14	18	6	
Dehydration degree	Mild	15	22	13	0.05
	Moderate	17	26	4	
	Severe	2	12	2	
Diarrhea duration (days)		0.95 ± 0.97	1.45 ± 1.11 ^a^	2.94 ± 1.86 ^b,c^	<0.01
Maximum number of stools/day		2.11 ± 2.57	5.15 ± 2.97 ^a^	4.05 ± 1.90 ^b,c^	<0.01
Vomiting duration (days)		1.25 ± 0.87	0.83 ± 0.56 ^a^	2.84 ± 1.86 ^b,c^	<0.01
Maximum vomiting episodes/day		3.85 ± 0.87	4.59 ± 0.56	1.26 ± 1.40 ^b,c^	<0.01
Maximum body temperature (°C)		37.42 ± 1.11	38.05 ± 0.99 ^a^	38.11 ± 1.17	0.02
Vesikari score		11.65 ± 2.25	13.32 ± 2.68 ^a^	10.74 ± 3.50 ^c^	<0.01
Modified Vesikari score		10.59 ± 2.09	12.14 ± 2.14 ^a^	11.16 ± 3.11	0.01

Legend: *—for each three-parameter mean comparison, the non-parametric Kruskal–Wallis test was used and Chi-square test was applied for contingency table analysis; ^a^—group 1 vs. group 2: *p* < 0.05; ^b^—group 1 vs. group 3: *p* < 0.05; ^c^—group 2 vs. group 3: *p* < 0.05; SD—standard deviation; *p* < 0.05—statistically significant.

**Table 2:** Comparison of patients’ paraclinical characteristics, based on gastroenteritis etiology.

Parameter	Group 1 (*n* = 34); Mean ± SD	Group 2 (*n* = 57); Mean ± SD	Group 3 (*n* = 19); Mean ± SD	*p* Value
pH	7.31 ± 0.09	7.32 ± 0.12	7.37 ± 0.11 ^c^	0.07 *
Na (mmol/L)	138.1 ± 3.65	140.5 ± 8.05	138.3 ± 6.44	0.49 *
K (mmol/L)	4.42 ± 0.88	4.41 ± 0.90	4.46 ± 0.89	0.94 **
HCO_3_ (mEq/L)	13.96 ± 8.85	14.12 ± 5.20	16.39 ± 5.65	0.42 **
Lactate (mmol/L)	2.42 ± 1.49	2.34 ± 2.59	1.37 ± 0.85 ^b,c^	0.01 *
Base excess (mmol/L)	−10.26 ± 7.63	−10.73 ± 7.89	−7.832 ± 6.63	0.29 **
Blood glucose (mg/dL)	93.12 ± 31.73	97.38 ± 38.34	88.63 ± 17.2	0.78 *
Leukocyte (/µL)	11,211 ± 5911	11,615 ± 5734	14,433 ± 4060 ^b,c^	0.03 *
Neutrophils (/µL)	6297 ± 5094	6327 ± 4155	7467 ± 4200	0.31 *
Lymphocytes (/µL)	3587 ± 3011	4019 ± 2655	5396 ± 2430 ^b,c^	0.01 *
Monocytes (/µL)	968.3 ± 908.7	1317 ± 873.5 ^a^	1406 ± 669 ^b^	<0.01 *
Eosinophils (/µL)	23.94 ± 58.08	65.64 ± 149.8	75.74 ± 101 ^b^	0.03 *
Basophils (/µL)	5.06 ± 5.99	38.85 ± 45.68 ^a^	39.95 ± 32.67 ^b^	<0.01 *
Platelets (/µL)	356,242 ± 175,028	420,627 ± 172,353 ^a^	488,053 ± 197,861 ^b^	0.01 *
MPV (fL)	9.78 ± 1.07	9.31 ± 1.01 ^a^	9.18 ± 1.53 ^b^	0.02 *
Hgb (g/dL)	11.32 ± 1.54	11.43 ± 1.84	11.27 ± 1.56	0.72 *
Htc (%)	34.01 ± 4.59	34.23 ± 4.19	32.19 ± 6.32	0.26 *
MEV (fL)	75.88 ± 8.72	75.94 ± 8.55	75.15 ± 10.27	0.90 *
ESR (mm/h)	20.46 ± 22.15	12.88 ± 17.04 ^a^	32.28 ± 35.08 ^c^	<0.01 *
CRP (mg/L)	14.35 ± 29.29	25.21 ± 42.31	49.47 ± 68.20 ^b,c^	0.01 *
AST (U/L)	42.03 ± 37.37	46.91 ± 22.64 ^a^	35.30 ± 12.28 ^c^	0.01 *
ALT (U/L)	27.07 ± 34.17	34.91 ± 31.17 ^a^	21.63 ± 10.88 ^c^	<0.01 *
GGT (U/L)	22.79 ± 25.57	22.02 ± 16.53	26.42 ± 26.39	0.35 *
Urea (mg/dL)	32.98 ± 26.23	33.64 ± 34.37	25.61 ± 27.60	0.25 *
Creatinine (mg/dL)	0.38 ± 0.25	0.50 ± 0.37	0.57 ± 0.70	0.11 *

Legend: *—for the three-parameter mean comparison, the non-parametric Kruskal–Wallis test was used; **—for the three-parameter mean comparison, the parametric Brown—Forsythe ANOVA test was used; ^a^—group 1 vs. group 2: *p* < 0.05; ^b^—group 1 vs. group 3: *p* < 0.05; ^c^—group 2 vs. group 3: *p* < 0.05; AST—aspartate aminotransferase; ALT—alanine aminotransferase; CRP—C-reactive protein; ESR—erythrocyte sedimentation rate; GGT—gamma glutamyl transferase; Hgb—hemoglobin; Htc—hematocrit; MEV—mean erythrocyte volume; MPV—mean platelet volume; SD—standard deviation; *p* < 0.05—statistically significant.

**Table 3:** Linear regression analysis between age, dehydration degree, paraclinical parameters and Vesikari/modified Vesikari scores.

Parameter	vs. Vesikari Score (r^2^ Value, *p* Value)	vs. Modified Vesikari Score (r^2^ Value, *p* Value)
Age	r^2^ = 0.005, *p* = 0.44	r^2^ = 0.006, *p* = 0.38
Dehydration degree (mild/moderate/severe)	r^2^ = 0.27, *p* < 0.01	r^2^ = 0.06, *p* < 0.01
pH	r^2^ = 0.06, *p* < 0.01	r^2^ = 0.01, *p* = 0.23
Lactate	r^2^ = 0.002, *p* = 0.64	r^2^ = 0.001, *p* = 0.67
HCO_3_	r^2^ = 0.09, *p* < 0.01	r^2^ = 0.02, *p* = 0.11
Base excess	r^2^ = 0.08, *p* < 0.01	r^2^ = 0.02, *p* = 0.08
Blood glucose	r^2^ = 0.02, *p* = 0.09	r^2^ = 0.01, *p* = 0.22
Na	r^2^ = 0.006, *p* = 0.38	r^2^ < 0.01, *p* = 0.87
K	r^2^ = 0.001, *p* = 0.65	r^2^ = 0.01, *p* = 0.87
Leukocyte	r^2^ <0.01, *p* = 0.93	r^2^ <0.01, *p* = 0.69
Neutrophil	r^2^ = 0.003, *p* = 0.54	r^2^ = 0.001, *p* = 0.71
Lymphocyte	r^2^ = 0.01, *p* = 0.26	r^2^ = 0.007, *p* = 0.36
Monocyte	r^2^ = 0.006, *p* = 0.40	r^2^ = 0.007, *p* = 0.36
Platelet	r^2^ = 0.01, *p* = 0.28	r^2^ = 0.03, *p* = 0.05
Hgb	r^2^ = 0.04, *p* = 0.02	r^2^ = 0.05, *p* = 0.01
Htc	r^2^ = 0.007, *p* = 0.37	r^2^ = 0.01, *p* = 0.28
ESR	r^2^ = 0.01, *p* = 0.23	r^2^ = 0.03, *p* = 0.06
CRP	r^2^ = 0.05, *p* = 0.01	r^2^ = 0.08, *p* < 0.01
AST	r^2^ = 0.07, *p* < 0.01	r^2^ = 0.05, *p* = 0.01
ALT	r^2^ = 0.04, *p* = 0.02	r^2^ = 0.01, *p* = 0.16
GGT	r^2^ = 0.02, *p* = 0.62	r^2^ = 0.02, *p* = 0.64
Urea	r^2^ = 0.13, *p* < 0.01	r^2^ = 0.05, *p* = 0.01
Creatinine	r^2^ = 0.01, *p* = 0.16	r^2^ = 0.004, *p* = 0.51

Legend: AST—aspartate aminotransferase; ALT—alanine aminotransferase; CRP—C-reactive protein; ESR—erythrocyte sedimentation rate; GGT—gamma glutamyl transferase; Hgb—hemoglobin; Htc—hematocrit; *p* < 0.05—statistically significant.

**Table 4:** Receiver operator characteristic (ROC) analysis of the Vesikari score for distinguishing between mild, moderate and severe dehydration.

Parameter	AUC (95% CI)	Cut-Off	Sensitivity (95% CI)	Specificity (95% CI)	*p* Value
Mild vs. moderate dehydration	0.70	>11.5	67.35% (53.38–78.79%)	61.22% (47.25–63.57%)	<0.01
Moderate vs. severe dehydration	0.78	>13.5	73.33% (48.05–89.10%)	63.27% (49.27–75.33%)	<0.01

Legend: AUC—area under curve; CI—confidence interval.

**Table 5:** Receiver operator characteristic (ROC) analysis of the modified Vesikari score for distinguishing between mild, moderate and severe dehydration.

Parameter	AUC (95% CI)	Cut-Off	Sensitivity (95% CI)	Specificity (95% CI)	*p* Value
Mild vs. moderate dehydration	0.58	>11.5	48.98% (35.58–62.53%)	63.27% (49.27–75.33%)	0.15
Moderate vs. severe dehydration	0.60	>13.5	60.00% (35.75–80.18%)	73.47% (59.74–83.79%)	0.22

Legend: AUC—area under curve; CI—confidence interval.

## Data Availability

The raw data supporting the conclusions of this article will be made available by the authors on request.
